# Correlation Between Lip Prominence and Orthodontic Incisor Repositioning Within an Aesthetic Triangle Framework

**DOI:** 10.3390/medicina62030556

**Published:** 2026-03-17

**Authors:** Sorana Maria Bucur, Eugen Silviu Bud, Mioara Decusară, Dana Cristina Bratu, Mariana Păcurar

**Affiliations:** 1 Department of Dentistry, Faculty of Medicine, “Dimitrie Cantemir” University of Târgu Mureș, 3-5 Bodoni Sandor Str., 540545 Târgu Mureș, Romania; bucursoranamaria@gmail.com; 2 Department of Orthodontics, Faculty of Dental Medicine, George Emil Palade University Medicine, Pharmacy, Science, and Technology of Târgu Mureș, 38 Gh. Marinescu Str., 540139 Târgu Mureș, Romania; mariana.pacurar@umfst.ro; 3 Department of Dentistry, Faculty of Medicine and Pharmacy, “Dunărea de Jos” University of Galați, 47 Domnească Str., 800008 Galați, Romania; 4 Department of Orthodontics II, Orthodontic Research Centre, Faculty of Dental Medicine, “Victor Babeș” University of Medicine and Pharmacy of Timișoara, 300041 Timișoara, Romania; bratu.cristina@umft.ro

**Keywords:** aesthetic triangle, lip prominence, orthodontics, incisor inclination, facial aesthetics, soft tissue response, profile analysis

## Abstract

*Background and Objectives*: Accurate prediction of lip prominence changes following orthodontic treatment remains challenging because traditional profile analyses rely on isolated reference lines that do not account for combined nasal and chin morphology. The aesthetic triangle framework integrates these structures and may provide a more comprehensive evaluation of lip position. *Materials and Methods*: This correlative clinical study evaluated 82 orthodontic patients undergoing bimaxillary incisor repositioning. Lateral cephalograms and standardized profile photographs were obtained before and after treatment. Lip position was assessed relative to the aesthetic triangle boundaries, and dentoalveolar changes were quantified using standard incisor measurements. Lip thickness was also analyzed as a potential modulating factor. *Results*: Mandibular incisor inclination demonstrated a moderate positive correlation with anterior displacement of the lower lip within the aesthetic triangle (Pearson r = 0.45, *p* < 0.01). Multiple linear regression analysis confirmed IMPA as a significant predictor of lower lip migration (β = 0.41), explaining approximately 21% of the observed variance (R^2^ = 0.21). In contrast, maxillary incisor inclination (U1–SN) showed weaker and statistically inconsistent associations with upper lip position. Compartment analysis revealed that approximately 32% of patients exhibited anterior migration of the lower lip from the posterior to the central aesthetic triangle compartment following treatment. These findings suggest that mandibular incisor inclination exerts a measurable influence on lower lip prominence, whereas upper lip positional changes appear to be less directly related to maxillary incisor variables. *Conclusions*: The aesthetic triangle provides a clinically meaningful framework for interpreting orthodontic soft-tissue changes as spatial migration rather than isolated linear measurements. Lower lip prominence responds predictably to dentoalveolar mechanics, whereas upper lip position also depends on soft tissue morphology.

## 1. Introduction

Facial aesthetics play a central role in social perception, self-esteem, and the demand for orthodontic and facial aesthetic treatment. Among facial components, the lips represent one of the most influential aesthetic units due to their prominence, dynamic function, and close anatomical relationship with underlying dentoalveolar and skeletal structures [1,2,3,4,5]. Their position contributes substantially to profile harmony and is strongly associated with perceived facial attractiveness and balance [3,4,5]. Consequently, accurate evaluation of lip prominence remains a critical component of orthodontic diagnosis and treatment planning.

Comprehensive lip assessment encompasses multiple interrelated factors, including morphology, functional activity, posture at rest, and degree of sagittal prominence [5,6,7]. The etiology of lip prominence is multifactorial, involving soft-tissue characteristics, skeletal relationships, and dentoalveolar support. Soft-tissue variables such as lip thickness, volume, tonicity, and elasticity significantly influence lip projection independent of skeletal position [1,3,5,8]. Additionally, variations in craniofacial morphology, particularly vertical growth patterns and lower anterior facial height, may modify lip posture by altering perioral soft-tissue tension and spatial relationships [3,9]. These complex interactions underscore the importance of integrated assessment approaches capable of capturing the combined influence of structural and functional determinants.

Dentoalveolar variables constitute a primary mechanical determinant of lip position. The sagittal position and inclination of the incisors provide direct support to the lips and substantially influence the soft-tissue profile [10,11]. Proclination and protrusion of the incisors generally increase lip prominence, whereas excessive retroclination may contribute to lip retrusion and flattening of the lower facial profile [10,11,12]. However, the magnitude of soft-tissue response to dentoalveolar repositioning varies considerably among individuals, reflecting the modulating role of lip morphology and biomechanical properties [5,6].

To accurately quantify dentoalveolar changes influencing lip support, standardized cephalometric parameters are commonly used to evaluate incisor position and inclination. Measurements such as U1–NA distance and U1–SN angulation assess maxillary incisor protrusion and axial inclination relative to cranial reference structures, while L1–NB distance and IMPA evaluate mandibular incisor sagittal position and inclination relative to the mandibular plane [10,11,12,13]. These variables were selected because they directly reflect the mechanical relationship between incisors and labial soft tissues and have been consistently demonstrated to correlate with changes in lip prominence following orthodontic treatment [10,11,14]. Their inclusion allows objective quantification of dentoalveolar support and facilitates analysis of its influence on lip migration within the aesthetic triangle framework.

To quantify lip prominence, several cephalometric reference lines have been proposed, including the Esthetic line (E-line), Steiner’s S-line, and the subnasale–soft tissue pogonion (Sn–Pog’) line [8,12,13,14]. These analyses relate lip position to key facial landmarks and provide normative reference values widely used in clinical practice. Nevertheless, the diagnostic reliability of isolated reference lines is influenced by numerous variables, including nasal projection, chin prominence, growth pattern, sex, and ethnicity [15,16]. Consequently, reliance on a single linear reference may lead to inconsistent interpretation of lip prominence across different facial morphologies.

To address these limitations, the aesthetic triangle concept was introduced as a composite diagnostic framework integrating the E-line and the Sn–Pog’ line into a bounded spatial zone for evaluating lip prominence [17]. Rather than defining lip position relative to a single reference line, this framework considers the spatial relationship of the lips to both the nasal and chin structures simultaneously. Lips positioned within the triangle are generally regarded as balanced relative to the facial profile, whereas those located anterior or posterior to its boundaries may be perceived as protrusive or retrusive, respectively [17]. Importantly, this approach allows lip position to be interpreted as placement within an aesthetic zone rather than as an isolated linear measurement.

Despite its conceptual advantages and clinical appeal, the aesthetic triangle has been applied primarily as a descriptive diagnostic tool. Empirical evidence remains limited regarding how orthodontic dentoalveolar changes influence lip position within this framework. In particular, little is known about whether incisor inclination and sagittal repositioning result in predictable migration of the lips between aesthetic triangle compartments, or whether upper and lower lips respond differently to dentoalveolar mechanics when evaluated within this integrated aesthetic context.

Improved understanding of these relationships is essential for enhancing diagnostic accuracy and predicting soft-tissue outcomes in orthodontic treatment [1,2,3,4,5,6,7,8]. Such knowledge may assist clinicians in distinguishing cases in which dentoalveolar correction alone can meaningfully influence lip prominence from those in which soft-tissue morphology limits aesthetic improvement.

Therefore, the present study aimed to evaluate changes in upper and lower lip prominence within the compartments of the aesthetic triangle and to determine their relationship with maxillary and mandibular incisor inclination and sagittal position following orthodontic treatment.

## 2. Materials and Methods

### 2.1. Study Design and Sample

This correlative clinical study was conducted on a sample of 82 orthodontic patients undergoing treatment involving controlled bimaxillary incisor repositioning. The study design aimed to evaluate the relationship between dentoalveolar incisor movement and changes in lip prominence within the aesthetic triangle framework.

Ethical approval No. 2709/27 December 2023 was obtained by the Ethics Committee of the George Emil Palade University of Medicine, Pharmacy, Science, and Technology of Târgu Mureș, before data collection. Written informed consent was secured from all participants. Patients were selected to minimize the potential influence of active craniofacial growth on soft tissue measurements.

Inclusion criteria comprised patients aged between 16 and 30 years with complete permanent dentition up to the second molars and no history of orthodontic treatment.

Exclusion criteria included previous orthognathic surgery, craniofacial syndromes, significant facial asymmetry, facial trauma, or soft tissue pathology affecting the lips.

### 2.2. Clinical Records

Standardized lateral cephalometric radiographs and profile photographs were obtained for each patient before and after orthodontic treatment. All records were acquired with the patient in natural head position (NHP), the mandible in rest position, and the lips in repose, to ensure consistency in soft tissue evaluation.

Cephalograms were taken with teeth in maximum intercuspation using the same radiographic equipment and settings for all patients. Profile photographs were captured under standardized lighting conditions with the patient standing upright and focusing on a distant point to maintain reproducible head posture.

All records were digitized and analyzed using calibrated orthodontic cephalometric software (Open-Ortho, version 1.1.3).

### 2.3. Aesthetic Triangle Construction and Lip Classification

The aesthetic triangle compartments were used to categorize lip position relative to the E-line, allowing both continuous millimetric measurements and categorical assessment of compartment migration following orthodontic treatment [17].

Lip prominence was evaluated using the aesthetic triangle concept as a composite diagnostic framework integrating two profile reference lines.

The anterior boundary of the aesthetic triangle was defined by the Esthetic line (E-line), constructed by joining the pronasale (Prn) to soft tissue pogonion (Pog’) [8,12].

The posterior boundary of the aesthetic triangle was defined by the subnasale–soft tissue pogonion (Sn–Pog’) line, thereby excluding nasal projection from posterior lip assessment [14].

Upper and lower lip positions were assessed relative to the anterior and posterior boundaries of the aesthetic triangle and classified into one of three triangle compartments:

- Posterior to the aesthetic triangle, when the lip was positioned posterior to the Sn–Pog’ line;
- Within the aesthetic triangle, when the lip was positioned between the Sn–Pog’ line and the E-line;
- Anterior to the aesthetic triangle, when the lip was positioned anterior to the E-line.

The esthetic triangle is divided by the Cp–Pog’ line into posterior (1) and anterior (2) compartments, with the posterior zone generally considered the most aesthetically favorable.

Pre-treatment to post-treatment changes were interpreted as anterior or posterior migration of the lips between aesthetic triangle compartments, allowing assessment of the direction and magnitude of soft tissue response to dentoalveolar incisor movement.

This classification enabled evaluation of lip prominence as a spatial relationship within an aesthetic zone rather than as an isolated linear measurement.

### 2.4. Dentoalveolar Measurements

Dentoalveolar incisor position and inclination were assessed on lateral cephalograms using standardized angular and linear measurements.

Maxillary incisor position and inclination were evaluated using:

U1–NA distance (mm) to assess sagittal protrusion.

U1–SN angle (degrees) to assess incisor inclination relative to the cranial base.

Mandibular incisor position and inclination were evaluated using:

L1–NB distance (mm) to assess sagittal protrusion.

IMPA (degrees) to assess incisor inclination relative to the mandibular plane.

These variables were selected because they represent standardized orthodontic indicators of incisor support for the lips and are routinely used to evaluate dentoalveolar protrusion.

### 2.5. Soft Tissue Thickness Assessment

Upper and lower lip thickness were measured on lateral cephalometric radiographs to evaluate their potential influence on soft-tissue response to dentoalveolar incisor repositioning. Lip thickness was defined as the perpendicular linear distance between the external lip surface and the corresponding labial surface of the incisors at the level of maximum lip prominence. Specifically, upper lip thickness was measured from the most anterior point of the upper lip soft tissue (labrale superius region) to the labial surface of the maxillary incisor crown along a line perpendicular to the long axis of the incisor. Lower lip thickness was measured analogously from the most anterior point of the lower lip soft tissue (labrale inferius region) to the labial surface of the mandibular incisor [6,8].

All measurements were performed on digitally calibrated cephalograms using orthodontic cephalometric analysis software. To minimize measurement variability, all landmarks were identified by the same trained examiner under standardized magnification conditions. These measurements were included as potential moderating variables influencing the magnitude of lip migration within the aesthetic triangle framework.

### 2.6. Statistical Analysis

Descriptive statistics, including means and standard deviations, were calculated for all dentoalveolar and soft tissue variables. Normality of data distribution was assessed using the Shapiro–Wilk test and graphical inspection of Q–Q plots. Because the variables did not show significant deviation from normality, parametric tests were applied.

Pearson correlation coefficients were used to assess associations between incisor inclination and sagittal position and changes in lip position relative to the aesthetic triangle boundaries.

Paired comparisons were performed to evaluate pre-treatment and post-treatment changes in dentoalveolar and soft tissue measurements.

Multiple linear regression analysis was performed to identify predictors of lip migration within the aesthetic triangle. Incisor inclination, sagittal incisor position, and lip thickness were entered as independent variables. Model assumptions, including linearity, homoscedasticity, and absence of multicollinearity, were evaluated before analysis.

Because the analyses were exploratory and based on predefined orthodontic variables derived from established theoretical models, adjustments for multiple comparisons were not applied to preserve statistical power. Therefore, results were interpreted with emphasis on effect size and clinical relevance rather than statistical significance alone. Statistical significance was set at *p* < 0.05.

A post hoc power analysis was conducted to determine whether the sample size was sufficient to detect clinically meaningful associations between dentoalveolar variables and lip migration. Based on the observed moderate correlation between mandibular incisor inclination (IMPA) and lower lip positional change (r ≈ 0.46) in a sample of 82 patients, the estimated statistical power exceeded 0.80 at a significance level of α = 0.05. These findings indicate that the study sample provided adequate power to detect moderate associations between orthodontic incisor variables and soft-tissue positional changes within the aesthetic triangle framework.

### 2.7. Methodological Rationale

The aesthetic triangle was selected as the primary evaluative framework because it integrates nasal and chin reference structures into a bounded spatial zone, allowing lip prominence to be interpreted as a positional relationship rather than an isolated linear measurement. This approach reduces dependence on single reference lines that may be distorted by individual variation in nasal projection or chin prominence. By classifying lip position into posterior, central, and anterior triangle compartments, treatment-related soft tissue changes can be assessed as directional migration within an aesthetic zone, thereby enhancing clinical interpretability and relevance to orthodontic treatment planning.

### 2.8. Orthodontic Treatment Protocol

Orthodontic treatment was performed using fixed appliance therapy with pre-adjusted edgewise brackets (0.022-inch slot). All patients underwent treatment aimed at controlled sagittal repositioning of maxillary and mandibular incisors.

Both extraction and non-extraction treatment protocols were included in the sample, depending on the individual orthodontic diagnosis and space requirements. However, in all cases, the treatment objective involved dentoalveolar incisor repositioning without orthognathic surgical intervention.

Biomechanical protocols were standardized to ensure controlled incisor movement. Alignment and leveling were followed by working archwires designed to achieve controlled incisor proclination or sagittal repositioning where required by the treatment plan.

Anchorage control was maintained using conventional intra-arch mechanics, including posterior anchorage units reinforced by stainless steel archwires. No skeletal anchorage devices were used in the analyzed cases.

Although minor variations in individual treatment mechanics were present, the fundamental therapeutic objective across all patients involved controlled bimaxillary incisor repositioning, allowing analysis of the associated soft-tissue response.

### 2.9. Measurement Reliability

To evaluate measurement reliability, 20 randomly selected cephalograms (approximately 25% of the sample) were reanalyzed two weeks after the initial assessment by the same examiner. Intra-examiner reliability was evaluated using intraclass correlation coefficients (ICC).

All evaluated variables demonstrated excellent reliability, with ICC values exceeding 0.90. Measurement error was further assessed using Dahlberg’s formula, confirming minimal random error in landmark identification and linear measurements.

These findings indicate high reproducibility of the cephalometric and soft-tissue measurements used in the present study.

## 3. Results

### 3.1. Dentoalveolar Changes Relevant to Aesthetic Triangle Assessment

Orthodontic treatment produced significant dentoalveolar changes affecting both maxillary and mandibular incisor position and inclination (Table 1). Maxillary incisors demonstrated an increase in sagittal prominence and proclination, as reflected by increased U1–NA distance and U1–SN angulation. Similarly, mandibular incisors exhibited increased protrusion and inclination, evidenced by increased L1–NB distance and IMPA values. These changes were accompanied by a marked reduction in interincisal angulation, indicating coordinated bimaxillary incisor repositioning.

**Table 1:** Dentoalveolar changes associated with aesthetic triangle assessment following orthodontic treatment (*n* = 82). Data are presented as mean ± SD. *p*-values were calculated using paired *t*-tests. Statistical significance was set at *p* < 0.05.

Parameter	Initial Mean ± SD	Final Mean ± SD	Direction
Maxillary incisor protrusion (U1–NA, mm)	0.66 ± 2.40	3.10 ± 2.25	Forward
Maxillary incisor inclination (U1–SN, °)	89.91 ± 6.99	104.02 ± 4.52	Proclination
Mandibular incisor protrusion (L1–NB, mm)	2.29 ± 2.40	4.83 ± 2.05	Forward
Mandibular incisor inclination (IMPA, °)	89.85 ± 6.90	102.00 ± 8.05	Proclination
Interincisal angle (°)	149.00 ± 11.81	122.30 ± 9.44	Decrease

These dentoalveolar changes provided the mechanical basis for subsequent soft-tissue adaptation and were therefore analyzed in relation to lip position within the aesthetic triangle framework.

**Figure 1:**
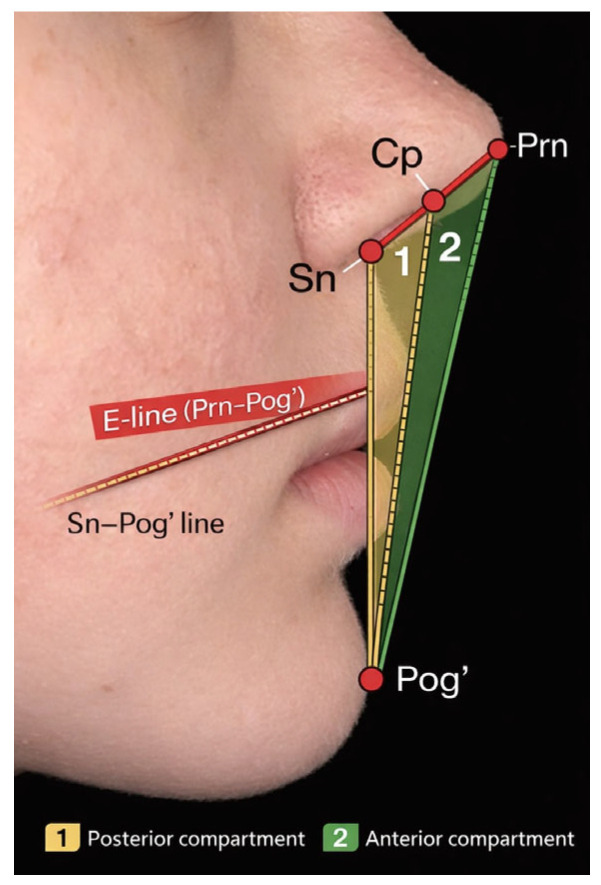
Evaluation of lip prominence within the aesthetic triangle framework. In natural head position, lip projection is assessed relative to an aesthetic triangle defined by the Sn–Pog’ line (posterior boundary) and the Prn–Pog’ esthetic line (E-line; anterior boundary). The Cp–Pog’ line divides the triangle into posterior (1) and anterior (2) compartments. Abbreviations: Sn = subnasale; Cp = columella point; Prn = pronasale; Pog’ = soft-tissue pogonion.

Lip position was interpreted within the aesthetic triangle framework (Figure 1). The triangle was further subdivided by the Cp–Pog’ line into anterior and posterior compartments.

### 3.2. Distribution of Lip Position Within Aesthetic Triangle Compartments

When lip position was interpreted within the aesthetic triangle framework, the Esthetic line (E-line) represented the anterior boundary of the triangle, while the subnasale–pogonion (Sn–Pog’) line defined its posterior boundary.

At baseline, both upper and lower lips were predominantly positioned within the posterior region of the aesthetic triangle, indicating a tendency toward relative lip retrusion. Following orthodontic treatment, changes in lip position relative to the anterior boundary of the triangle were observed.

The upper lip demonstrated minimal sagittal change relative to the E-line, remaining largely within the posterior compartment of the aesthetic triangle. Mean upper lip position relative to the E-line showed no clinically relevant anterior migration, indicating limited migration within the triangle despite dentoalveolar incisor advancement.

In contrast, the lower lip exhibited a consistent anterior shift relative to the E-line following treatment. This movement reflected a migration from a more posterior triangle position toward the central compartment of the aesthetic triangle. Although the lower lip remained, on average, posterior to the E-line, its position moved closer to the anterior boundary of the triangle, indicating an increase in perceived prominence within the aesthetic zone. The magnitude of this change corresponded to a small-to-moderate effect size, suggesting that even modest sagittal lip migration may contribute to clinically perceptible improvement in profile balance.

These findings demonstrate differential soft tissue response of the upper and lower lips within the aesthetic triangle following dentoalveolar incisor repositioning (Table 2).

**Table 2:** Upper and lower lip position relative to the anterior boundary of the aesthetic triangle (Esthetic line) before and after treatment (*n* = 82).

Parameter	Initial Mean ± SD (mm)	Final Mean ± SD (mm)	Triangle-Based Interpretation
Upper lip to E-line	−5.63 ± 2.93	−5.69 ± 2.32	Stable within the posterior compartment
Lower lip to E-line	−3.81 ± 2.83	−3.17 ± 2.84	Anterior migration toward the central compartment

In addition to linear measurements relative to the E-line, categorical analysis of lip position within the aesthetic triangle compartments was performed to evaluate treatment-related positional shifts (Table 3).

**Table 3:** Distribution of lip position within aesthetic triangle compartments before and after orthodontic treatment (*n* = 82).

Lip Position Category	Pre-Treatment (*n*)	Post-Treatment (*n*)	Observed Migration
Posterior to triangle	54	38	Reduction following treatment
Within triangle	28	40	Increase due to anterior migration
Anterior to triangle	0	4	Small number of anterior shifts

Approximately 32% of patients demonstrated anterior migration of the lower lip from the posterior to the central aesthetic triangle compartment following treatment, indicating clinically observable improvement in sagittal lip prominence for a substantial proportion of the sample.

### 3.3. Incisor Inclination as a Predictor of Aesthetic Triangle Compartment Migration

Pearson correlation analysis demonstrated a moderate positive association between mandibular incisor inclination and anterior migration of the lower lip within the aesthetic triangle. In particular, increases in the incisor mandibular plane angle (IMPA) were moderately correlated with forward movement of the lower lip relative to the triangle boundaries. Mandibular incisor protrusion measured by the L1–NB distance also showed a positive correlation with lower lip migration, although the magnitude of this association was slightly weaker.

To further quantify the predictive strength of dentoalveolar variables influencing lip position within the aesthetic triangle, Pearson correlation coefficients and multiple linear regression analyses were performed (Table 4).

**Table 4:** Correlation and regression analysis of dentoalveolar predictors of lip migration within the aesthetic triangle (*n* = 82).

Predictor Variable	Pearson r	β (Standardized)	95% CI for β	R^2^ Contribution	Interpretation
IMPA (Mandibular incisor inclination)	0.46	0.41	0.19–0.62	0.21	Moderate positive association
L1–NB distance	0.38	0.33	0.11–0.54	0.18	Mild–moderate association
U1–SN angle	0.19	0.14	−0.04–0.31	0.05	Weak association
U1–NA distance	0.16	0.12	−0.05–0.29	0.04	Weak association

Abbreviations: IMPA—Incisor mandibular plane angle; L1–NB—mandibular incisor protrusion relative to NB line; U1–SN—maxillary incisor inclination relative to SN plane; U1–NA—maxillary incisor protrusion relative to the NA line.

Mandibular incisor inclination (IMPA) demonstrated the strongest relationship with lower lip migration, with a moderate correlation coefficient (r = 0.46). Regression analysis confirmed IMPA as a significant predictor of anterior lower lip migration (β = 0.41), accounting for approximately 21% of the variance in lower lip positional change. Similarly, mandibular incisor protrusion (L1–NB) showed a mild to moderate association with lower lip migration (r = 0.38; β = 0.33), contributing approximately 18% of the explained variance.

In contrast, maxillary incisor variables demonstrated weaker and less consistent relationships with upper lip position relative to the aesthetic triangle boundaries. Maxillary incisor inclination (U1–SN) showed only a weak correlation with upper lip positional change (r = 0.19), while sagittal protrusion of the maxillary incisors (U1–NA) exhibited minimal predictive value (r = 0.16). Regression coefficients for these variables were small and confidence intervals included values close to zero, suggesting limited predictive influence.

Overall, these findings indicate that mandibular incisor inclination represents the most relevant dentoalveolar determinant of lower lip migration within the aesthetic triangle framework. The magnitude of the observed associations corresponds to a moderate effect size, supporting the clinical relevance of mandibular incisor mechanics in influencing lower lip prominence following orthodontic treatment.

### 3.4. Modulating Effect of Lip Thickness on Triangle-Based Lip Response

Upper and lower lip thickness measurements were evaluated to assess their influence on soft tissue response to dentoalveolar movement within the aesthetic triangle (Table 5).

**Table 5:** Changes in lip thickness as a modulating factor of aesthetic triangle response (*n* = 82).

Parameter	Initial Mean ± SD (mm)	Final Mean ± SD (mm)	Observed Effect
Upper lip thickness	12.67 ± 2.51	12.00 ± 2.39	Minimal change
Lower lip thickness	13.81 ± 2.48	12.56 ± 2.09	Decrease

The upper lip demonstrated relatively stable thickness values before and after treatment, which may partially explain the limited sagittal migration observed despite changes in maxillary incisor position. In contrast, a modest reduction in lower lip thickness was observed following treatment, coinciding with increased lower lip prominence within the triangle.

Correlation analysis indicated that thinner lips exhibited greater anterior migration within the aesthetic triangle for a given amount of dentoalveolar incisor movement, whereas thicker lips demonstrated a dampened response. This effect was more pronounced in the lower lip than in the upper lip.

These findings suggest that lip thickness acts as a modulating factor influencing the magnitude of triangle-based lip migration in response to orthodontic incisor repositioning.

### 3.5. Summary of Aesthetic Triangle Outcomes

Overall, orthodontic treatment involving bimaxillary incisor repositioning resulted in measurable changes in lip position when interpreted within the aesthetic triangle framework. While maxillary incisor advancement produced limited upper lip migration within the triangle, mandibular incisor repositioning was associated with consistent anterior migration of the lower lip toward the central aesthetic compartment.

The aesthetic triangle approach allowed these changes to be interpreted as directional movement within a defined aesthetic zone, rather than as isolated linear measurements, thereby enhancing the clinical relevance of the observed soft tissue responses.

## 4. Discussion

The present study evaluated lip prominence within the framework of the aesthetic triangle and investigated its relationship with dentoalveolar incisor repositioning following orthodontic treatment. The results indicate that orthodontic incisor proclination produces differential soft-tissue responses between the upper and lower lips when interpreted within a bounded aesthetic zone rather than relative to isolated cephalometric reference lines. Specifically, mandibular incisor advancement was consistently associated with anterior displacement of the lower lip toward the central compartment of the aesthetic triangle, whereas maxillary incisor proclination produced only limited migration of the upper lip despite substantial dentoalveolar change.

Although the aesthetic triangle has previously been described primarily as a diagnostic element in facial aesthetic assessment (Naini, 2021) [8], the present findings suggest that its clinical utility may extend beyond descriptive analysis. By evaluating the geometric configuration of the triangle in relation to biomechanical parameters such as incisor inclination and lip thickness, the current study provides clinical evidence supporting its potential prognostic relevance. In this context, the aesthetic triangle may serve not only as a tool for assessing lip position but also as a framework for anticipating soft-tissue responses to orthodontic tooth movement. Consequently, the concept may be interpreted as evolving from a purely diagnostic construct toward a predictive model capable of assisting clinicians in treatment planning and in estimating post-treatment lip profile changes.

### 4.1. Dentoalveolar Changes as the Mechanical Basis for Soft-Tissue Adaptation

The dentoalveolar changes observed in this study confirmed significant bimaxillary incisor proclination and sagittal advancement, creating the biomechanical conditions necessary for soft-tissue adaptation. Coordinated increases in U1–NA distance, U1–SN angulation, L1–NB distance, and IMPA were accompanied by a reduction in interincisal angulation, reflecting controlled dentoalveolar repositioning.

These findings are consistent with established orthodontic principles recognizing the incisors as primary determinants of labial support and soft-tissue profile [10,11]. Previous studies have demonstrated that changes in incisor inclination are closely associated with alterations in lip position, although the magnitude of response varies considerably among individuals due to differences in soft-tissue morphology and biomechanical properties [5,10]. Such variability highlights the importance of assessment frameworks capable of contextualizing dentoalveolar effects within broader facial structures [18,19].

### 4.2. Interpretation of Lip Prominence Within the Aesthetic Triangle

When lip position was interpreted within the aesthetic triangle framework, distinct patterns of soft-tissue adaptation became evident. The lower lip demonstrated consistent anterior migration toward the central triangle compartment, whereas the upper lip remained predominantly within the posterior compartment despite comparable dentoalveolar advancement [17].

This asymmetric response likely reflects anatomical and biomechanical differences between the upper and lower lips [3,8,20]. The lower lip is more directly supported by the mandibular incisors and therefore tends to follow dentoalveolar movement more closely. In contrast, the upper lip is influenced by additional structural factors, including nasal projection, philtral morphology, and perioral muscular tension, which may limit its sagittal responsiveness to incisor repositioning alone. Consequently, orthodontic mechanics may exert a stronger and more predictable influence on lower lip prominence than on upper lip position within the facial profile [3,5,8,19,20].

Previous investigations have similarly reported that the lower lip demonstrates greater positional responsiveness to orthodontic mechanics than the upper lip [10,11]. Furthermore, studies evaluating facial soft-tissue morphology have shown that lip projection is strongly influenced by the spatial relationship between nasal and chin structures, supporting the rationale for integrated assessment approaches [15,16]. Within this context, the aesthetic triangle provides a framework that incorporates these anatomical relationships and allows lip position to be interpreted relative to both facial boundaries simultaneously [17]. Because nasal morphology and perioral muscular activity were not directly measured in the present study, these factors should be interpreted as potential contributors rather than definitive causal determinants.

### 4.3. Clinical Value of the Aesthetic Triangle Framework

A major strength of the present study lies in the application of the aesthetic triangle as an interpretive framework for evaluating orthodontic soft-tissue changes. Traditional analyses based on single reference lines, such as the E-line, may overemphasize numerical measurements without adequately contextualizing their aesthetic significance [16]. Because these reference lines are influenced by nasal projection and chin prominence, their isolated use may lead to inconsistent interpretation across different facial morphologies [15,16].

In contrast, the aesthetic triangle enables lip prominence to be assessed as spatial placement within a defined aesthetic zone bounded by nasal and chin landmarks [17]. Within this framework, anterior migration of the lower lip toward the central triangle compartment may be interpreted as movement toward improved profile balance rather than simply as numerical proximity to a reference line. This spatial perspective enhances clinical interpretability and facilitates more meaningful evaluation of treatment outcomes.

### 4.4. Modulating Role of Soft-Tissue Morphology and Lip Thickness

The present findings further demonstrate the modulating influence of lip thickness on soft-tissue response to dentoalveolar movement. Thinner lips exhibited greater anterior migration within the aesthetic triangle for a given degree of incisor advancement, whereas thicker lips showed a dampened positional response.

These observations are consistent with previous studies indicating that lip thickness and biomechanical properties influence the extent to which incisor movement is translated into profile change [5,6,11]. Thicker or less elastic soft tissues may absorb dentoalveolar migration without substantial alteration in sagittal position, while thinner tissues tend to follow incisor movement more closely. This modulating effect was particularly evident in the lower lip, further supporting the concept that upper and lower lips should not be assumed to respond uniformly to orthodontic mechanics [21].

Although the mean linear changes in lip position were relatively modest, previous aesthetic perception studies have demonstrated that sagittal lip migrations of approximately 1–2 mm may already influence perceived facial profile harmony [22]. Therefore, the magnitude of lower lip migration observed in the present study may represent a clinically meaningful improvement in facial balance for selected patients.

### 4.5. Clinical Implications

From a clinical perspective, the present findings highlight the importance of comprehensive soft-tissue diagnosis in orthodontic treatment planning. Evaluation within the aesthetic triangle allows clinicians to determine not only whether lip position changes following treatment but also whether those changes occur within an aesthetically meaningful zone [23,24].

For patients presenting with lower lip retrusion within the posterior triangle compartment, mandibular incisor advancement may significantly improve lip prominence and profile balance. In patients with posteriorly positioned upper lips and thick or tonically resistant soft tissues, the expectations of aesthetic improvement solely from incisor proclination should be tempered. In such cases, adjunctive treatment approaches, including orthognathic or soft-tissue procedures, may be required to achieve optimal aesthetic outcomes [25,26].

### 4.6. Limitations and Future Directions

Certain limitations should be acknowledged. The present study was limited to two-dimensional profile analysis, which does not fully capture three-dimensional soft-tissue morphology or volumetric changes. Three-dimensional imaging modalities may provide additional insight into the spatial dynamics of lip adaptation [27]. Furthermore, the analysis focused primarily on sagittal lip prominence, without addressing vertical or dynamic aspects of lip function [28,29].

Future research incorporating three-dimensional assessment, longitudinal follow-up, and patient-reported aesthetic outcomes may further validate the clinical applicability of the aesthetic triangle framework. Additionally, evaluation across diverse ethnic groups and facial morphologies would enhance generalizability and refine normative reference ranges.

Although the aesthetic triangle provides a useful integrative framework for evaluating sagittal lip prominence, it should be interpreted as a complementary diagnostic tool rather than a standalone determinant of facial aesthetics.

## 5. Conclusions

Within the limitations of this retrospective cephalometric study, mandibular incisor inclination demonstrated a moderate association with anterior displacement of the lower lip within the aesthetic triangle framework. Regression analysis indicated that mandibular incisor inclination accounted for a portion of the variability in lower lip positional change, whereas maxillary incisor inclination showed limited predictive value for upper lip displacement.

The aesthetic triangle model provided a clinically interpretable framework for evaluating soft-tissue positional changes relative to dentoalveolar movements. However, the magnitude of lip displacement observed suggests that perioral soft tissues may respond variably to dentoalveolar changes, likely influenced by additional factors such as lip thickness, nasal morphology, and muscular dynamics that were not directly assessed in this study.

Future investigations incorporating three-dimensional imaging and direct evaluation of soft-tissue characteristics may further clarify the biomechanical relationship between incisor positioning and facial soft-tissue adaptation.

## Data Availability

The raw data supporting the conclusions of this article will be made available by the authors on request.
